# The Literature of Chemoinformatics: 1978–2018

**DOI:** 10.3390/ijms21155576

**Published:** 2020-08-04

**Authors:** Peter Willett

**Affiliations:** Information School, University of Sheffield, Sheffield S1 4DP, UK; p.willett@sheffield.ac.uk

**Keywords:** bibliometrics, cheminformatics, chemoinformatics, scientometrics

## Abstract

This article presents a study of the literature of chemoinformatics, updating and building upon an analogous bibliometric investigation that was published in 2008. Data on outputs in the field, and citations to those outputs, were obtained by means of topic searches of the *Web of Science Core Collection*. The searches demonstrate that chemoinformatics is by now a well-defined sub-discipline of chemistry, and one that forms an essential part of the chemical educational curriculum. There are three core journals for the subject: The *Journal of Chemical Information and Modeling*, the *Journal of Cheminformatics*, and *Molecular Informatics*, and, having established itself, chemoinformatics is now starting to export knowledge to disciplines outside of chemistry.

## 1. Introduction

Increasing use is being made of bibliometric methods to analyze the published academic literature, with studies focusing on, e.g., author productivity, the articles appearing in a specific journal, the characteristics of bibliographic frequency distributions, new metrics for research evaluation, and the citations to publications in a specific subject area inter alia (e.g., [1,2,3,4,5,6]). There have been many bibliometric studies of various aspects of chemistry over the years, with probably the earliest such study being the famous 1926 paper by Lotka in which he discussed author productivity based in part on an analysis of publications in *Chemical Abstracts* [7]. There have, however, been only a few publications to date that have applied bibliometric methods to quantitative structure–activity relationships (QSARs) and to chemoinformatics, the foci of this special issue of the *International Journal of Molecular Sciences*. Willett and co-workers have studied some of the journals most closely connected with these topics [8,9,10], but there have been only two bibliometric articles that have studied QSAR and chemoinformatics as subjects in themselves (rather than journals about these subjects) in any detail [11,12].

In the first of these, Willett [11] found that the *Journal of Chemical Information and Modeling* was the core journal for chemoinformatics for the period 1998–2006, but with many significant papers published in journals whose principal focus was molecular modelling, QSAR, or more general aspects of chemistry. The discipline was international in scope, and many of the most cited papers were descriptions of widely used chemoinformatics software packages]. Li et al. [12] studied QSAR publications over the period 1993 to 2012. They found that the number of articles per year quadrupled from 1993 to 2006 but plateaued thereafter, with articles on molecular descriptors and modelling important for drug design and articles on model validation and reliability important for the environmental sciences. Their analysis mirrored the chemoinformatics study, in that there were contributions to the literature from a wide range of countries and the *Journal of Chemical Information and Modeling* was again the largest source of articles from amongst the journals that were analyzed in their study. This short communication provides a bibliometric overview of the chemoinformatics literature up to the end of 2018 as represented in the *Web of Science Core Collection* (hereafter WoS) database from Clarivariate Analytics, and hence represents an update to, and an extension of, that presented in [11]. An appendix contains a brief introduction to the literature for those new to the field of chemoinformatics.

## 2. Results and Discussion

### 2.1. Outputs

**Figure 1:**
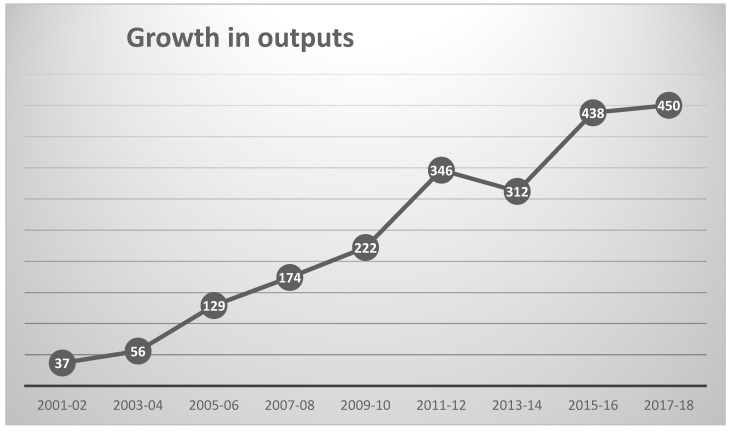
Growth in annual chemoinformatics outputs since 2001.

The WoS Topic search (see Section 3) identified 2195 outputs, a total that represents a substantial increase on the 197 outputs analyzed in 2008 [11]; it is, however, only a very small fraction of the 19,214 pre-2019 outputs in a search for QSAR, let alone the 45,697 for the related field of bioinformatics. The growth in publication during the current century is shown in Figure 1 (which encompasses all but the 31 outputs published prior to 2001). The earliest mention of “chemical informatics” was in 1978 [13], with the earliest mentions of “cheminformatics” and “chemoinformatics” occurring in 1997 [14] and 1999 [15], respectively. It can be seen from the figure that the initial steady growth in the literature appears to have started to level off, as noted by Li et al. for QSAR [12] whereas publications in bioinformatics continue to increase year on year.

The 2195 outputs came from a total of 740 different sources, though no less than 513 of these provided only a single contribution. The 10 most productive sources are listed in Table 1, where the numbers of outputs for the *Journal of Chemical Information and Modeling* and for *Molecular Informatics* include those published in the previous incarnations of the journals (*Journal of Chemical Information and Computer Sciences* for the former; and first *Quantitative Structure–Activity Relationships* and then *QSAR and Combinatorial Science* for the latter) and where the IF column contains the 2018 impact factors for the eight sources where these are available (the other two sources are not journals and hence do not have journal IFs). The most productive source is the published abstracts of the twice-yearly national meetings of the American Chemical Society (ACS). There were 220 of these, i.e., 10% of all of the items considered here; however, such conference presentations are very infrequently cited, contributing just 9 from the total of 25,188 citations discussed in Section 2.2. With the exception of the ACS abstracts and *Methods in Molecular Biology* (which is a monograph series), all of the other sources in the table are academic journals; this is also the case for all but one of the next 25 sources when they are ranked in order of decreasing productivity, the sole exception being the 11th-ranked *Lecture Notes in Computer Science*, which is a monograph series that contains conference proceedings. The ACS abstracts and then *Journal of Chemical Information and Modeling* were also the two most productive sources in [11], but they have been joined at the top of the ranking here by two other sources that have clearly established themselves as core journals for the field. These are the *Journal of Cheminformatics*, which started publication in 2009, and *Molecular Informatics*, which started publication in 2010 as a successor to *QSAR and Combinatorial Science*. The latter journal has changed not only its name but also its subject focus since 100 of the 109 articles listed in Table 1 come from *Molecular Informatics*, against just 9 from its two previous QSAR-focused incarnations.

All but one of the sources in Table 1 are what one might expect for a discipline that is heavily involved in drug discovery and design (and this also applies to the great majority of the next 25 sources, which include, e.g., *ChemMedChem*, *Expert Opinion on Drug Discovery*, *Journal of Medicinal Chemistry*, *Journal of Molecular Graphics and Modelling*, and *Molecular Diversity*). The exception in Table 1 is the *Journal of Chemical Education*, and its presence here demonstrates that chemoinformatics has now become an accepted part of the chemical curriculum, with an entire issue of the journal devoted to the subject in 2016 [16] and with the first of these 69 articles only appearing in 2005. In a similar vein, the subject’s increasing recognition as an established sub-discipline in chemistry is demonstrated by the fact that the 2195 outputs included 8 books and 109 book chapters, with the first of these again only appearing in 2005.

The outputs’ authors come from a total of 80 different countries, but the great majority of the outputs (93% of them) involve just the 10 countries listed in Table 2. The same 10 countries are also the most productive whether we consider outputs from 2009 onwards (the first year in which there was more than 100 outputs) or outputs up to and including 2008. Eight of these countries also figure in the 10 most productive countries in the WoS research area of chemistry for the period 1978–2018: The only differences are that Canada and Switzerland in Table 1 are replaced by Spain and Russia, so that national productivity in chemoinformatics would appear to closely mirror that in chemistry more generally. The USA’s position at the top of the ranking in Table 2 is hardly unexpected given its leadership in most areas of science (including chemistry overall, where it has almost twice as many publications as the People’s Republic of China (PRC), the next most productive nation). That said, its prominence in the table here is due in part at least to the inclusion of the 220 ACS abstracts mentioned previously since 163 of them have USA authors.

The 10 most productive organizations are listed in Table 3. As would be expected for an academic research field, eight of these are university groups, headed by acknowledged leaders in the field (e.g., Bender and Glen at Cambridge, Tropsha at North Carolina, and Wild at Indiana) and universities continue to be by far the most prominent type if one considers, e.g., the 50 most productive organizations. Two, however, are not, with 34 outputs coming from Collaborations in Chemistry and 31 from the Novartis Institutes for Biomedical Research (with a further 10 coming from Novartis Pharma AG). The first is a company run by Ekins, who has made significant contributions to data sharing and to making pharmaceutical data more open, while the second is one of the world’s major pharmaceutical companies. While there are several governmental and professional organizations in the top 50 (e.g., the Chinese Academy of Sciences, the European Bioinformatics Institute, the Environmental Protection Agency, and the Royal Society for Chemistry), the only other commercial organization is AstraZeneca, another major pharmaceutical company. Such organizations emphasize the importance of chemoinformatics to the pharmaceutical industry.

### 2.2. Citations

**Figure 2:**
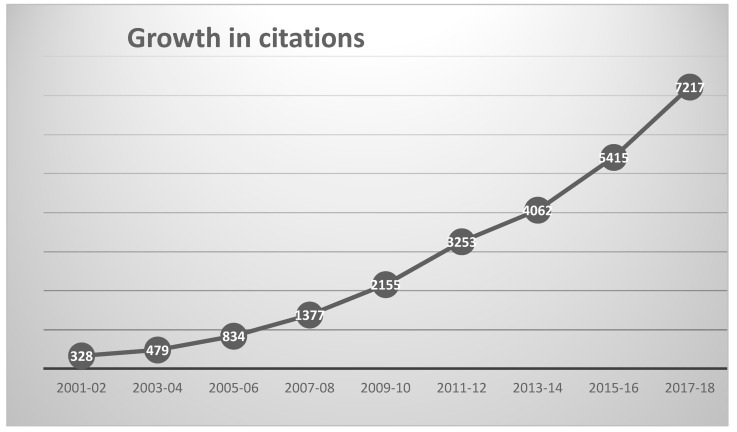
Growth in annual citations to chemoinformatics publications since 2001.

The 2195 chemoinformatics outputs had been cited by 25,188 outputs published in 5004 different sources up to the end of 2018, as shown in Figure 2. The 10 most heavily cited outputs are listed in Table 4, these together accounting for almost 20% of the total number of citations (93% of which came from journal articles).

It can be seen that the outputs in Table 4 have a very strong focus on databases, websites, or software for data analysis. This was also the case in the previous study [11] and this trend is very likely to continue to be the case given the current interest in data analysis and machine learning methods that require large amounts of data if they are to provide high levels of predictive performance. Thus, an article by Daina et al. [27] in *Scientific Reports* that introduced a website for ADME (absorption, distribution, metabolism, and excretion) prediction had already attracted 538 citations by May 2020, despite only being published in March 2017 (i.e., too late for it to have received sufficient citations by the end of 2018 to have been included in Table 4). The interest in machine learning is evidenced by the presence in Table 4 of the articles describing support vector machines and random forests, two of the leading types of software for this purpose, and by the very many outputs (500 of them as of May 2020) from the three core journals—*Journal of Chemical Information and Modeling*, *Journal of Cheminformatics*, and *Molecular Informatics*—that are retrieved in a WoS topic search for “machine learning”. Indeed, each issue of *Journal of Chemical Information and Modeling* now has a sub-section given over specifically to articles on machine learning and deep learning.

Table 4 also shows that only two of the outputs in the table (those by O’Boyle et al. [17] and by Svetnik et al. [20]) were published in the sources listed in Table 1, i.e., while there is a well-defined core to the literature of chemoinformatics, many significant contributions to the field are published elsewhere. Some of these contributions, moreover, appear in very high-impact journals (e.g., the 2018 impact factors for *Nature Genetics* and *Nucleic Acids Research* are 25.455 and 11.142 against 3.966 and 4.154 for *Journal of Chemical Information and Modeling* and *Journal of Cheminformatics*), further emphasizing the broadening recognition of the importance of chemoinformatics.

The citations come from a total of 5004 different sources, with 5 of the 10 most frequently citing journals (Journal of Chemical Information and Modeling, Journal of Cheminformatics, Molecular Informatics, Journal of Computer-Aided Molecular Design, and Current Topics in Medicinal Chemistry) included in the set of the 10 most productive sources shown in Table 1. This is hardly surprising, and similar comments apply if citations are considered only to the 405 chemoinformatics papers in the 3 core journals. However, it is also worth noting that *PLoS ONE* (the first of the new generation of open-access megajournals [28,29]) is one of the 10 most frequently citing journals for each of these three core journals, and *Scientific Reports*, another prominent megajournal, is also one of the 10 most frequently citing journals for *Journal of Cheminformatics*, which is the only fully open-access journal in Table 1. Both of these megajournals cover all aspects of science, hence demonstrating the increasing visibility of chemoinformatics to the broader scientific community. However, some of the other citing journals are in specific disciplines that are very far removed from chemistry, let alone the areas of molecular modelling and drug design that are the focus of this special issue. This behavior is an example of what Cronin and Pearson described as a knowledge export, where a discipline, A, is said to export knowledge when a citation is made from an article in another discipline to an article in A [30]. The number of citations to articles in A can hence be used to assess its influence on scholarship in general [31], and this was investigated here by means of the subject categories used in the WoS database.

Each journal (and hence each article in each journal) in the WoS database is assigned to one or more of 255 subject categories, and it is hence possible to explore the extent of knowledge exports from the chemoinformatics cohort by considering the subject categories of the citing articles. One would expect the most frequent categories to come from the chemical, biological, and computer science categories and this is indeed the case, e.g., 4836 of the citing outputs were assigned the chemistry medicinal category, and 3911 and 3469 to the biochemistry molecular biology and computer science interdisciplinary applications categories, respectively. What is perhaps surprising is that at least one citation has come from no less than 216 of the 255 categories, many of which would seem on first glance to have little or no obvious relationship to chemoinformatics, but which do indeed have a relationship when examined more closely. For example, articles from the categories fisheries [32] and music [33] in the journals *Canadian Journal of Fisheries and Aquatic Sciences* and *Journal of New Music Research* are amongst the many on applications of machine learning that cite the article by Svetnik et al. in Table 4 on the use of random forests; indeed, the 834 citations for this article have come from publications in no less than 130 different categories, a number that is significantly greater than for any of the other highly cited articles in the table.

There are many other non-obvious examples of knowledge export, such as an article from the surgery category in *Pediatric Surgery International* on medical education in Africa [34] that cites one by Wild and Wiggins on distance learning in chemoinformatics [35], an article from the mycology category in *Fungal Genetics and Biology* on building a database of fungal natural products [36] that cites one by Heller et al. on the InChI (International Chemical Identifier) notation [37], and an article from the otorhinolaryngology category in *European Archives of Oto-Rhino-Laryngology* on the evaluation of a software planning tool for cochlear implant surgery [38] that cites one by Todeschini et al. on chemical similarity coefficients [39].

In conclusion, this paper reviewed the literature of chemoinformatics as delineated in the *Web of Science Core Collection* up to and including 2018. While some of the findings reported here are analogous to those in a previous bibliometric study that was published in 2008 [11], at least three differences are apparent. First, while the *Journal of Chemical Information and Modeling* is still the most productive journal, the *Journal of Cheminformatics* and *Molecular Informatics* have clearly established themselves and can now be spoken of in the same breath when considering the core journals for the field. Next, chemoinformatics has become recognized as a well-defined sub-discipline that forms an essential part of the chemical educational curriculum, and one that is increasingly covered in the monograph literature. Finally, having established itself within the discipline of chemistry, it is now starting to export knowledge to a wide range of other disciplines.

## 3. Materials and Methods

Articles on chemoinformatics may not, of course, contain that particular word; but those that do contain it may be assumed (with a fair degree of probability) to contain material about that subject. Publications for analysis were hence identified in May 2020 in exactly the same way as in [11] with a topic search of the WoS database using the query chemoinformatics OR cheminformatics OR “chemical informatics” (where a topic search covers the title, abstract, and keyword fields) for items published up to the end of 2018. Of these three search terms, “chemical informatics” was by far the least common, retrieving less than 5% of the total of 2195 items, with the remainder shared approximately equally between “chemoinformatics” (1071 items) and “cheminformatics” (1059 items). Journal articles comprised by far the largest proportion of the outputs (78%), but there was also meeting abstracts, papers from conference proceedings, book chapters, etc.

## 4. Appendix

It will be clear from the above that there is now an extensive chemoinformatics literature. For those new to the field, the best single introduction to the subject is probably the book by Leach and Gillet [40], with a more recent, but far larger, coverage being presented in two books edited by Engel and Gasteiger [41,42]. Chen [43] and Willett [44] describe the subject’s historical development, and there are now many excellent reviews that provide introductions to specific topics in chemoinformatics, e.g., conformational analysis [45], data mining [46], library design [47], machine learning [48], molecular similarity [49], patent information systems [50], pharmacophore analysis [51], reaction databases [52], scaffold hopping [53], structure representations [54], text mining [55], and virtual screening [56] inter alia.

## Figures and Tables

**Table 1:** Sources producing the largest numbers of chemoinformatics outputs.

Source	Outputs	IF
*Abstracts of Papers of the American Chemical Society*	220	
*Journal of Chemical Information and Modeling*	185	3.996
*Journal of Cheminformatics*	111	4.154
*Molecular Informatics*	109	2.375
*Journal of Chemical Education*	69	1.763
*Current Topics in Medicinal Chemistry*	41	3.442
*Journal of Computer-Aided Molecular Design*	41	3.250
*Combinatorial Chemistry & High Throughput Screening*	39	1.503
*Chemical Biology & Drug Design*	32	2.256
*Methods in Molecular Biology*	31	

**Table 2:** Nations producing the largest numbers of chemoinformatics outputs.

Nation	Outputs
United States of America	822
United Kingdom	312
Germany	230
People’s Republic of China	128
France	112
India	109
Switzerland	95
Canada	89
Japan	83
Italy	68

**Table 3:** Organizations producing the largest numbers of chemoinformatics outputs.

Organization	Outputs
University of Cambridge	58
University of North Carolina	51
University of Sheffield	41
Universidade do Porto	38
Indiana University	35
Collaborations in Chemistry	34
Universidad Nacional Autónoma de México	34
Novartis Institutes for Biomedical Research	31
University of Strasbourg	26
University of Bonn	25

**Table 4:** Chemoinformatics articles attracting the largest numbers of citations.

Output	Citations
O’Boyle N.M. et al. Open Babel: An open chemical toolbox. *J. Cheminform.* **2011**, *3*, 33, doi:10.1186/1758-2946-3-33. [17]	1526
Wishart D.S. et al. DrugBank: a comprehensive resource for in silico drug discovery and exploration. *Nucleic Acids Res.* **2006**, *34*, D668-D672, doi:10.1093/nar/gkj067. [18]	1344
Scherf, U. et al. A gene expression database for the molecular pharmacology of cancer. *Nat. Genet.* **2000**, *24*, 236-244, doi:10.1038/73439. [19]	1065
Svetnik, V. et al. Random forest: A classification and regression tool for compound classification and QSAR modeling. *J. Chem. Inf. Comput. Sci.* **2003**, *43*, 1947-1958, doi:10.1021/ci034160g. [20]	834
Xia, J. et al. MetaboAnalyst: a web server for metabolomic data analysis and interpretation. *Nucleic Acids Res.* **2009**, *37*, W652-W660, doi:10.1093/nar/gkp356. [21]	689
Allen, F.H.; Motherwell, W.D.S. Applications of the Cambridge Structural Database in organic chemistry and crystal chemistry. *Acta Crystallogr. B Struct. Sci. Cryst. Eng. Mater.* **2002**, *58*, 407-422, doi:10.1107/S0108768102004895. [22]	502
Dix, D.J. et al. The ToxCast program for prioritizing toxicity testing of environmental chemicals. *Toxicol. Sci.* **2007**, *95*, 5-12, doi:10.1093/toxsci/kfl103. [23]	420
Burbidge, R. et al. Drug design by machine learning: support vector machines for pharmaceutical data analysis. *Comput. Chem.* **2001**, *26*, 5-14, doi:10.1016/S0097-8485(01)00094-8. [24]	403
Koch, M.A. et al. Charting biologically relevant chemical space: A structural classification of natural products (SCONP). *Proc. Natl. Acad. Sci. U.S.A.* **2005**, *102*, 17272-17277, doi:10.1073/pnas.0503647102. [25]	387
Hopkins, A.L. et al. Can we rationally design promiscuous drugs? *Curr. Opin. Struct. Biol.* **2006**, *16*, 127-136, doi:10.1016/j.sbi.2006.01.013. [26]	332

## Abbreviations

WoS	*Web of Science Core Collection*
QSAR	Quantitative structure–activity relationship
